# Number of Previous Strokes and the Association With Clinical Outcomes of Patients With Atrial Fibrillation: Longitudinal Data From the GLORIA‐AF Registry

**DOI:** 10.1161/JAHA.124.038448

**Published:** 2025-01-16

**Authors:** Steven Ho Man Lam, Giulio Francesco Romiti, Bernadette Corica, Tommaso Bucci, Brian Olshansky, Tze‐Fan Chao, Menno V. Huisman, Gregory Y. H. Lip, the GLORIA‐AF Investigators

**Affiliations:** ^1^ Liverpool Centre for Cardiovascular Sciences at University of Liverpool Liverpool John Moores University and Liverpool Heart & Chest Hospital Liverpool UK; ^2^ Department of Translational and Precision Medicine Sapienza–University of Rome Rome Italy; ^3^ Division of Cardiology, Department of Medicine University of Iowa Iowa City IA USA; ^4^ Division of Cardiology, Department of Medicine Taipei Veterans General Hospital Taipei Taiwan; ^5^ Institute of Clinical Medicine, and Cardiovascular Research Center National Yang Ming Chiao Tung University Taipei Taiwan; ^6^ Department of Thrombosis and Hemostasis Leiden University Medical Center Leiden the Netherlands; ^7^ Danish Center for Health Services Research, Department of Clinical Medicine Aalborg University Aalborg Denmark

**Keywords:** atrial fibrillation, cardiovascular risk, number of stroke events, Atrial Fibrillation

## Abstract

**Background:**

Patients with atrial fibrillation (AF) who suffered a previous stroke are at increased risk of recurrent thromboembolic events and other major outcomes. The impact of the number of stroke episodes on the natural history of patients with AF is still unclear.

**Methods and Results:**

Using data from the international, multicenter, and prospective GLORIA‐AF (Global Registry on Long‐Term Oral Antithrombotic Treatment in Patients With Atrial Fibrillation) Registry Phase III, we categorized patients with a recent diagnosis of non‐valvular AF according to the number of previous strokes (either 0, 1, or ≥2 episodes). We analyzed use of oral anticoagulants through multiple logistic regression model, and risk of major outcomes using multiple Cox‐regression models; our primary outcome was all‐cause death. Among 21 223 patients (mean age: 70.2±10.3 years; 44.9% female) included, 2251 (10.6%) had a previous history of stroke, and 216 (1.0%) had ≥2 or more strokes. Oral anticoagulants were used in ≥80% of patients regardless of the numbers of previous stroke, although those with 1 (versus >1) prior stroke showed lower odds of receiving oral anticoagulants (odds ratio [95% CI]: 0.83 [0.73–0.94]). During 3‐years follow‐up, the risk of all‐cause mortality increased with the number of previous strokes (hazard ratio [95% CI]: 1.46 [1.28–1.67] and 2.43 [1.79–3.29] for 1 versus 0 and ≥2 versus 0 previous strokes, respectively). Similar results were observed for other secondary outcomes, including thromboembolism, but not for major bleeding.

**Conclusions:**

History of stroke still represents a key risk factor in patients with AF. Patients who suffered more than 1 episodes of stroke had significantly worse prognosis and further efforts may be required to improve their clinical outcomes.

**Nonstandard Abbreviations and Acronyms**
GLORIA‐AF: global registry on long‐term oral antithrombotic treatment in patients with atrial fibrillation
NOAC: non‐vitamin K antagonist oral anticoagulant
OAC: oral anticoagulant
VKA: vitamin K antagonist

**Clinical Perspectives**

**What Is New?**

- This study highlights that 10% of newly diagnosed patients with atrial fibrillation have a history of stroke, with 1% experiencing multiple stroke episodes.
- Patients with more strokes have greater comorbidity and higher mortality risk.

**What Are the Clinical Implications?**

- The findings emphasize the need for personalized, aggressive stroke prevention strategies, including optimization of oral anticoagulation, particularly for patients with atrial fibrillation with multiple strokes and complex health profiles.

Despite reduction in thromboembolic events in patients with atrial fibrillation (AF) over the last few decades, stroke remains a consequence of AF^1^ and is responsible for substantial risk of death, disability and reduced quality of life.^2^, ^3^, ^4^ While the decline in the incidence of thromboembolic events can be ascribed to higher use of oral anticoagulants (OACs) in recent years, and particularly after the introduction of the non‐vitamin K antagonist oral anticoagulants (NOACs),^5^, ^6^ recurrent strokes still occur in patients with AF,^7^ despite appropriate use of OACs.^8^, ^9^, ^10^

Patients who suffer a stroke are at increased risk of other cardiovascular events, in the context of the “Stroke‐Heart Syndrome,”^11^, ^12^ which further complicates the prognosis of these patients. Indeed, patients with recurrent (and therefore, multiple) strokes may present with more complex health needs and challenges in the management, and require a more aggressive approach to reduce the risk of subsequent events. This has been acknowledged in a recent international consensus which advocated for a post‐stroke integrated care approach to reduce the risk of stroke recurrence, re‐hospitalization, and major cardiac events, ensure better functional capabilities, and improve overall prognosis of these patients.^13^ Therefore, epidemiological data on real‐world AF patients with multiple previous episodes of stroke are needed, to clarify the management, trajectories and outcomes of this high‐risk subgroup of patients.

In this study, we aimed to identify the relations between thromboembolic risk management and long‐term outcomes of patients with a recent diagnosis of AF, according to the number of previous stroke episodes reported at baseline.

## METHODS

Data supporting this study by the data contributors Boehringer Ingelheim, and were made and are available through Vivli, Inc. Access was provided after a proposal was approved by an independent review committee identified for this purpose and after receipt of a signed data sharing agreement.

### Study Design

We employed the data from the GLORIA‐AF (Global Registry on Long‐Term Oral Antithrombotic Treatment in Patients With Atrial Fibrillation) Registry phase III. Full details on the protocol, study design, study procedures and primary results of the GLORIA‐AF Registry had been published before.^14^, ^15^, ^16^, ^17^ In brief, GLORIA‐AF is a worldwide, multicenter, prospective registry program composed of 3 phases which aimed to evaluate the safety and effectiveness of dabigatran etexilate among patients with a recent diagnosis of AF (<3 months or <4.5 in Latin America). To be eligible for enrolment, patients must be 18 years or older, with a recent diagnosis of non‐valvular AF and a CHA_2_DS_2_‐VASc score ≥1. Detailed inclusion and exclusion criteria were published previously.^16^ Phase I was conducted before the approval of dabigatran with data collected only at baseline visit; in phase II, patients were followed for 2 years if they were treated with dabigatran at baseline. Phase III was performed between 2014 and 2016, and patients enrolled were followed for 3 years regardless of antithrombotic treatment received at baseline. Ethics approvals were received from local institutional review boards and written informed consents were provided by each patient included. The study was performed in accordance with the Declaration of Helsinki.

### Data Collection

Demographics and clinical data were collected using standardized electronic case report form across all sites worldwide. At baseline, investigator recorded clinical characteristics, including comorbidities and treatment, and whether patients had a previous history of stroke, the number of strokes suffered by the patient (either none, 1, or 2 or more episodes), and the type of the most recent stroke (either ischemic, hemorrhagic or unknown). In our primary analysis, we included all patients, regardless of the most recent stroke type; we also performed a sensitivity analysis, in which we focused on those patients who had an ischemic stroke as the latest stroke episode.

Data on antithrombotic treatment at baseline were also recorded, ie, whether the patient received oral anticoagulant (OAC) and the type of OAC (either vitamin‐K antagonist [VKA] or non‐vitamin‐K antagonist oral anticoagulant [NOAC]), antiplatelets or no antithrombotic treatment. Additionally, dose of NOAC received (either standard, reduced or other dose) was also collected.

### Study Outcomes

For this analysis, we considered all‐cause mortality as our primary outcome of interest. We also evaluated secondary outcomes including the occurrence of major adverse cardiovascular events (which included cardiovascular death, stroke, and myocardial infarction), cardiovascular death, recurrent stroke, thromboembolism (defined as the composite of stroke, transient ischemic attack, and other non‐central nervous system thromboembolism) and major bleeding (defined as a life‐threatening or fatal bleeding, symptomatic bleeding in a critical organ, or a bleeding associated with a hemoglobin reduction of ≥20 g/L or leading to ≥2‐unit of blood transfusion).

### Statistical Analysis

Continuous variables and categorical variables were presented as median and interquartile range (IQR) and count (percentage) respectively. Baseline characteristics between groups with different number of strokes were analyzed with Mann–Whitney U test, or chi‐squared teste for continuous variables and categorical variables respectively. Multiple adjusted logistic regression models were used to analyze the association between the number of episodes of stroke and the use of OAC. Models were adjusted for the components of CHA_2_DS_2_‐VASc score other than history of thromboembolic events (age <65, 65–75 or ≥75 years, sex, hypertension, diabetes, Heart Failure [HF], Coronary Artery Disease [CAD], and Peripheral Artery Disease [PAD]), as well as body mass index, type of AF (paroxysmal, persistent or permanent), region of recruitment and history of previous bleeding. Cox regression models were used to evaluate the association between number of stroke and the risks of major outcomes, with the same adjustment described above, and with further adjustment for the use of OAC.

For our primary outcome, we (i) reported Kaplan–Meier survival curves, and performed Log‐Rank test to evaluate the differences in the survival probabilities between patients according to the history of previous stroke, and (ii) modeled an interaction between the number of stroke episodes and key clinical characteristics (i.e., age, sex, history of CAD, HF, bleeding events, use and type of OAC, standard versus reduced dose of NOAC, and Asian ethnicity), to explore if the number of stroke episodes was heterogeneously associated with risk of all‐cause mortality across relevant subgroups. Finally, we performed a sensitivity analysis, restricted to patients who had an ischemic stroke as the latest (or unique) episode of previous stroke. All statistical analyses were performed with R version 4.3.1 (R Core Team 2020, Vienna, Austria), and a two‐sided *P*<0.05 was considered statistically significant.

## RESULTS

### Baseline Characteristics

A total of 21 223 patients with available data were included in this analysis (mean [SD]) age (years): 70.2 (10.3), 44.9% female. At baseline, 2251 (10.6%) patients had a previous history of stroke, of whom 2035 (9.6%) had one and 216 (1.0%) patients had ≥2 subsequent strokes. Baseline characteristics are described in Table 1.

**Table 1:** Baseline Characteristics of the Cohort and the Comparison Among Different Groups of Different Number of Previous Strokes

Variables	No previous stroke (*n*=18 972)	1 Previous stroke (*n*=2035)	≥2 Previous strokes (*n*=216)	*P* value
Age, mean (SD)	69.9 (10.4)	72.5 (9.7)	74.2 (8.9)	<0.001
<65 y	5009/18972 (26.4)	367/2035 (18.0)	33/216 (15.3)	
65–74 y	6915/18972 (36.4)	702/2035 (34.5)	69/216 (31.9)	
≥75 y	7048/18972 (37.1)	966/2035 (47.5)	114/216 (52.8)	
Female sex, n (%)	8573/18972 (47.5)	864/2035 (42.5)	94/216 (43.5)	0.058
BMI, median [IQR]	27.6 [24.5–31.6]	26.6 [24.0–30.1]	26.4 [23.7–29.7]	
Asian, n (%)	3607/17952 (20.1)	445/1894 (23.5)	45/216 (21.7)	0.002
Region, n (%)				
Europe	9018/18972 (47.5)	1137/2035 (55.9)	119/216 (55.1)	<0.001
North America	4739/18972 (25.0)	339/2035 (16.7)	31/216 (14.4)	
Asia	3724/18972 (19.6)	445/2035 (21.9)	45/216 (20.8)	
Other	1491/18972 (7.9)	114/2035 (5.6)	21/216 (9.7)	
AF type, n (%)				
Paroxysmal AF	10 658/18972 (56.2)	1174/2035 (57.7)	129/216 (59.7)	<0.001
Persistent AF	6558/18972 (34.6)	630/2035 (31.0)	58/216 (26.9)	
Permanent AF	1756/18972 (9.3)	231/2035 (11.4)	29/216 (13.4)	
Medical history, n (%)				
Hypertension	14 093/18931 (74.4)	1549/2029 (76.3)	184/216 (85.2)	<0.001
Heart failure	4208/18825 (22.4)	349/2011 (17.4)	39/214 (18.2)	<0.001
CAD	3504/18498 (18.9)	433/1995 (21.7)	38/207 (18.4)	0.011
Diabetes	4371/18972 (23.0)	492/2035 (24.2)	72/216 (33.3)	0.001
Hyperlipidemia	7258/18507 (39.2)	951/1995 (47.7)	111/209 (53.1)	<0.001
PAD	523/18841 (2.8)	82/2015 (4.1)	14/212 (6.6)	<0.001
COPD	1138/18771 (6.1)	122/2013 (6.1)	21/214 (9.8)	0.074
Previous bleeding	919/18621 (4.9)	183/1990 (9.2)	23/214 (10.7)	<0.001
Abnormal kidney function	324/18737 (1.7)	59/2011 (2.9)	5/211 (2.4)	0.001
Dementia	90/18774 (0.5)	27/2006 (1.3)	6/212 (2.8)	<0.001
History of cancer	1893/18701 (10.1)	205/2005 (10.2)	24/213 (11.3)	0.853
Ischemic stroke (most recent episode)	‐	1719/2035 (84.5)	201/216 (93.1)	‐
Risk scores				
CHA_2_DS_2_‐VASc, median [IQR]	3 [2–4]	5 [4–6]	5 [5–6]	<0.001
HAS‐BLED, median [IQR]	1 [1–2]	2 [2–3]	2 [2–3]	<0.001
Antithrombotic treatment, n (%)				
None	1231/18963 (23.0)	138/2033 (6.8)	9/216 (4.2)	0.001
Antiplatelets	2060/18963 (10.9)	267/2033 (13.1)	30/216 (13.9)	
NOAC	11 312/18963 (59.7)	1224/2033 (60.2)	121/216 (56.0)	
VKA	4360/18963 (23.0)	404/2033 (19.9)	56/216 (25.9)	
Dose of NOAC, n (%)				
Standard	7891/11312 (69.8)	812/1224 (66.3)	69/121 (57.0)	0.001
Reduced	3295/11312 (29.1)	391/1224 (31.9)	49/121 (40.5)	
Other	126/11312 (1.1)	21/1224 (1.7)	3/121 (2.5)	
Other treatments, n (%)				
ACE inhibitors	5613/18972 (29.6)	632/2035 (31.1)	72/216 (33.3)	0.199
ARB	4813/18972 (25.4)	452/2035 (22.2)	53/216 (24.5)	0.007
Statins	7944/18972 (41.9)	1437/2035 (70.6)	171/216 (79.2)	<0.001
Beta‐blockers	12 049/18972 (63.5)	1191/2035 (58.5)	119/216 (55.1)	<0.001

ACE indicates angiotensin‐converting enzyme; AF, atrial fibrillation; ARB, angiotensin receptor blocker; BMI, body mass index; CAD, coronary artery disease; COPD, chronic obstructive pulmonary disease; IQR, interquartile range; NOAC, non‐vitamin‐K antagonist oral anticoagulant; PAD, peripheral artery disease; and VKA, vitamin K antagonist.

Patients with previous strokes were more likely to be older and enrolled in Europe. They also had a higher prevalence of hypertension (85.2% versus 76.3% versus 74.4% in patients with 2 or more, 1, or 0 previous episodes of stroke, respectively; *P*<0.001). Similarly, there were differences between groups concerning diabetes, hyperlipidemia, chronic obstructive pulmonary disease, dementia and previous bleeding events. As expected, they also showed higher median CHA2DS2‐VASc scores (5,^5^, ^6^ 5^4^, ^5^, ^6^ and 3^2^, ^3^, ^4^ in patients with 2 or more, 1, or 0 previous episodes of stroke, respectively; *P*<0.001). Patients with higher number of previous strokes were also more likely to be treated with statins (79.2% versus 70.6% versus 41.9% in patients with 2 or more, 1, or 0 previous episodes of stroke, respectively; *P*<0.001), but were also less treated with beta‐blockers (*P*<0.001).

### Use of Oral Anticoagulants

**Figure 1:**
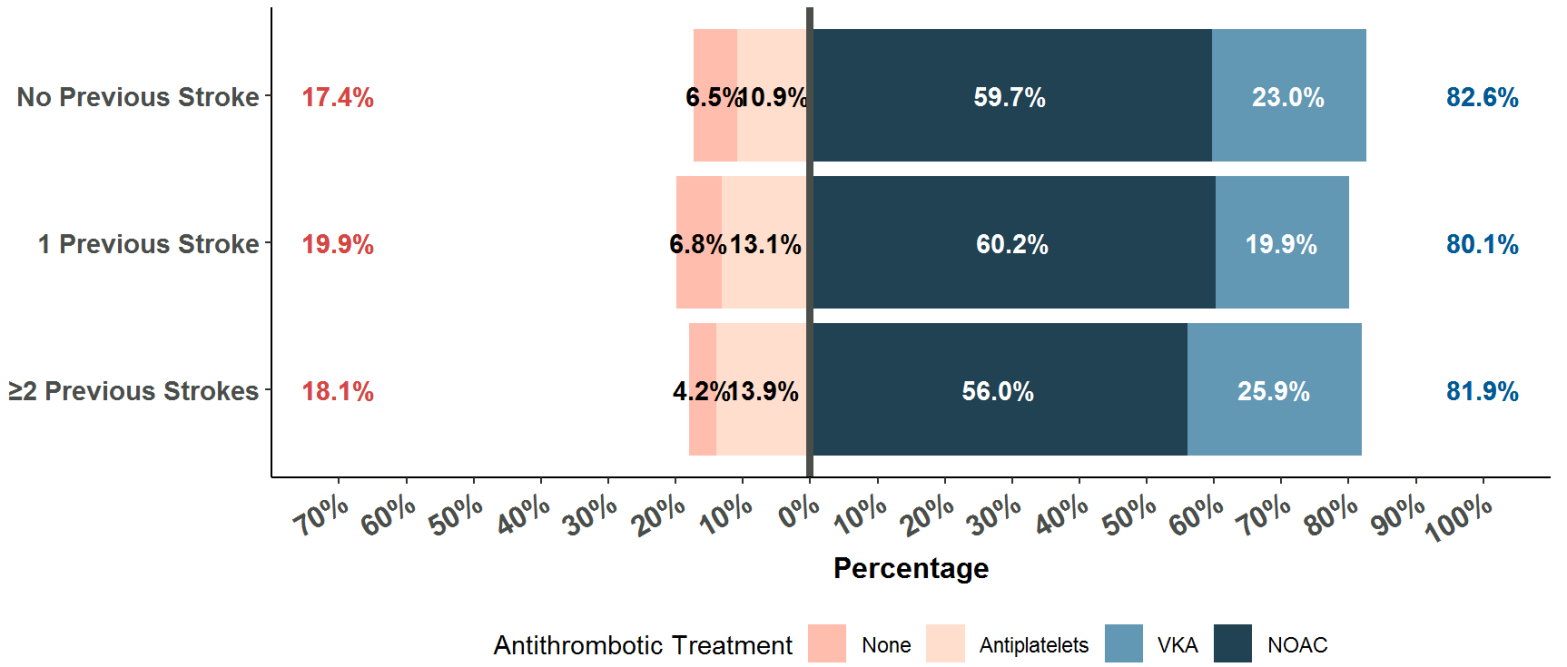
Use of antithrombotics according to the previous number of strokes. NOAC indicates non vitamin‐K antagonist oral anticoagulants; and VKA, Vitamin K antagonist.

A consistently high use of OACs was observed in our cohort at baseline, regardless of the previous strokes suffered; >80% of patients received OACs in each group of patients with different episodes of previous strokes. We observed a numerically higher proportion of patients receiving VKAs in those who had ≥2 strokes (25.9%; Figure 1), while standard doses of NOACs were more used in patients with 0 or 1 previous episodes of stroke (*P*=0.001; Table 1).

Multiple adjusted logistic regression analyses indicated that patients with 1 prior stroke had a marginally significant lower likelihood of OAC use (OR [95% CI]: 0.83 [0.73–0.94]), but when OAC was used there was a higher use of NOACs versus VKA (OR [95% CI]: 1.19 [1.05–1.34]), when compared with no prior stroke. These associations were not statistically significant in patients with ≥2 previous episodes of stroke (Figure S1).

### Incidence of Major Outcomes

**Figure 2:**
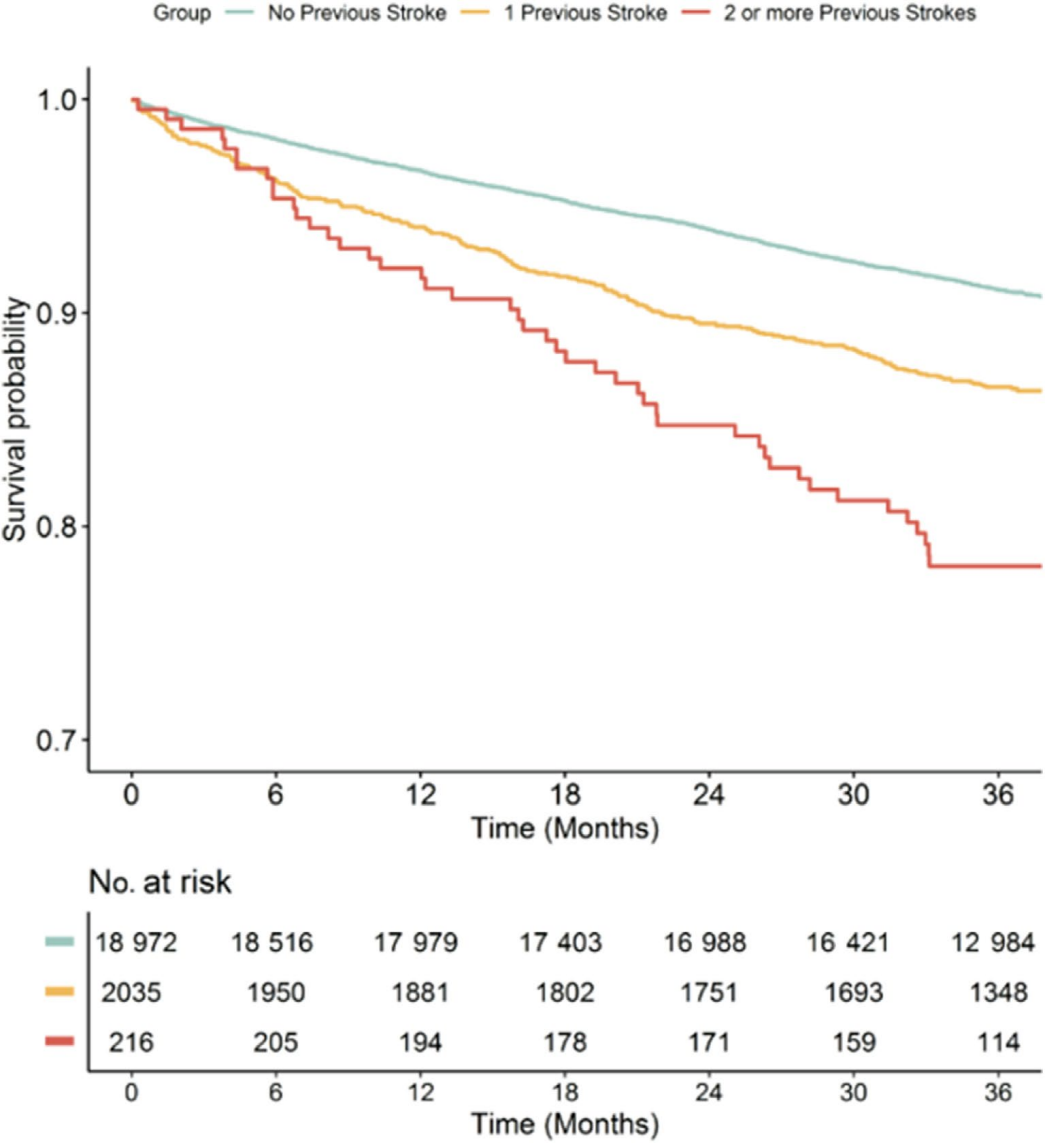
Survival curves according to the number of previous strokes for the primary outcome of all‐cause death. Log‐Rank *P*<0.001.

After 3 years of follow‐up, 1997 (9.4%) all‐cause deaths were observed; survival curves for all‐cause mortality were reported in Figure 2. Progressively increasing incidence of all‐cause mortality was observed as the number of previous episodes of stroke increased (Log‐rank *P*<0.001). The multiple adjusted Cox regression analysis showed that patients with 1 or ≥2 strokes were at greater risk of all‐cause death (hazard ratio [HR] [95% CI]: 1.46 [1.27–1.66] and 2.42 [1.79–3.29], respectively; *P*<0.001 for all; Table 2). Similar results were observed for all the other secondary outcomes (cardiovascular death, major adverse cardiovascular event, recurrent stroke, and thromboembolism), except for major bleeding, for which no statistically significant association was observed (Table 2). Table S1 reveals all the predictors of recurrent stroke in our cohort.

**Table 2:** Multiple Cox Regressions on Major Outcomes According to the Number of Previous Strokes

	No previous stroke (*n*=18 972)	1 Previous stroke (*n*=2035)	≥2 Previous strokes (*n*=216)
Primary outcome			
All‐cause death			
aHR [95% CI]	Reference	1.46 [1.28–1.67]*	2.43 [1.79–3.29]*
Secondary outcomes			
Cardiovascular death			
*aHR* [95% CI]	Reference	1.31 [1.03–1.65]*	2.67 [1.62–4.40]*
MACE			
*aHR* [95% CI]	Reference	1.59 [1.36–1.86]*	2.86 [2.02–4.05]*
Recurrent stroke aHR [95% CI]	Reference	2.07 [1.66–2.58]*	3.32 [2.04–5.42]*
Thromboembolism			
aHR [95% CI]	Reference	2.35 [1.96–2.83]*	3.82 [2.55–5.72]*
Major bleeding			
aHR [95% CI]	Reference	1.11 [0.87–1.42]	1.01 [0.48–2.13]

aHR indicates adjusted hazard ratio; MACE, major adverse cardiovascular events.

* Depicts statistically significant results at *P*<0.05 level.

### Subgroup Analyses

In the subgroup analyses, we did not observe any statistically significant interaction between numbers of previous stroke and key relevant clinical characteristics (age, sex, history of coronary artery disease, heart failure, or bleeding, Asian ethnicity, use and type of OAC, and standard versus reduced dose of NOAC). The result indicated that an increasing number of stroke episodes was consistently associated with a higher risk of all‐cause mortality during follow‐up across patients with different clinical characteristics (Table 3).

**Table 3:** Interaction Analysis on the Risk of the Primary Outcome (All‐Cause Death)

Subgroup		No. of previous stroke (vs 0)	aHR [95% CI]	*P value*	*P* for interaction
Age	<65 y	1 Previous stroke	1.30 [0.79–2.13]	0.306	0.651
		≥2 Previous strokes	2.69 [0.86–8.43]	0.089	
	65–74 y	1 Previous stroke	1.65 [1.27–2.15]	<0.001	
		≥2 Previous strokes	3.18 [1.86–5.43]	<0.001	
	≥75 y	1 Previous stroke	1.41 [1.20–1.66]	<0.001	
		≥2 Previous strokes	2.13 [1.44–3.15]	<0.001	
Sex	Men	1 Previous stroke	1.54 [1.30–1.83]	<0.001	0.144
		≥2 Previous strokes	3.04 [2.09–4.41]	<0.001	
	Women	1 Previous stroke	1.35 [1.10–1.67]	0.004	
		≥2 Previous strokes	1.71 [1.01–2.91]	0.047	
Coronary artery disease	No	1 Previous stroke	1.43 [1.22–1.68]	<0.001	0.760
		≥2 Previous strokes	2.54 [1.82–3.55]	<0.001	
	Yes	1 Previous stroke	1.51 [1.19–1.92]	0.001	
		≥2 Previous strokes	1.97 [0.93–4.16]	0.076	
Heart failure	No	1 Previous stroke	1.47 [1.25–1.73]	<0.001	0.988
		≥2 Previous strokes	2.43 [1.71–3.47]	<0.001	
	Yes	1 Previous stroke	1.44 [1.14–1.81]	0.002	
		≥2 Previous strokes	2.41 [1.32–4.38]	0.004	
Previous bleeding	No	1 Previous stroke	1.48 [1.28–1.70]	<0.001	0.849
		≥2 Previous strokes	2.43 [1.76–3.36]	<0.001	
	Yes	1 Previous stroke	1.30 [0.86–1.97]	0.213	
		≥2 Previous strokes	2.35 [0.96–5.78]	0.062	
OAC	No	1 previous stroke	1.66 [1.28–2.16]	<0.001	0.423
		≥2 Previous strokes	3.06 [1.67–5.60]	<0.001	
	Yes	1 Previous stroke	1.40 [1.20–1.63]	<0.001	
		≥2 Previous strokes	2.27 [1.59–3.23]	<0.001	
Asian ethnicity	Asian	1 Previous stroke	1.54 [1.10–2.15]	0.012	0.761
		≥2 Previous strokes	3.14 [1.54–6.40]	0.002	
	Non Asian	1 Previous stroke	1.44 [1.24–1.68]	<0.001	
		≥2 Previous strokes	2.38 [1.69–3.35]	<0.001	
NOAC vs VKA	VKA	1 Previous stroke	1.73 [1.34–2.24]	<0.001	0.195
		≥2 Previous strokes	2.21 [1.24–3.92]	0.007	
	NOAC	1 Previous stroke	1.29 [1.06–1.56]	0.011	
		≥2 Previous strokes	2.29 [1.46–3.57]	<0.001	
NOAC dose	Standard dose	1 Previous stroke	1.49 [1.15–1.92]	0.002	0.277
		≥2 Previous strokes	2.51 [1.30–4.87]	0.006	
	Reduced dose	1 Previous stroke	1.09 [0.81–1.47]	0.570	
		≥2 Previous strokes	2.05 [1.12–3.74]	0.020	

aHR, adjusted hazard ratio; OAC, oral anticoagulant.

### Sensitivity Analysis on Ischemic Stroke

We performed a sensitivity analysis, restricted to those patients who had an ischemic stroke as the latest (or unique) episode of previous stroke. Overall, we included 21 085 patients in this analysis, of which 1920 (9.1%) had previous ischemic stroke.

We observed consistent results in terms of the likelihood of receiving different anti‐coagulants (OAC, NOAC, and VKA) in the multivariable logistic regression analysis (Figure S2‐S3), as well as the risk of all‐cause death (HR [95% CI]: 1.49 [1.29–1.71] for patients with 1 previous stroke; (HR [95% CI]: 2.47 [1.81–3.37] for patients with 2 or more episodes of stroke) and secondary outcomes (Figure S4 and Table S2).

## DISCUSSION

In this analysis from a contemporary and prospective registry of patients with a recent diagnosis of AF, our principal findings are as follows: (1) up to 10% of patients with AF still present with a history of previous stroke at diagnosis, and 1 out of 10 of these patients had suffered 2 or more strokes; (2) patients with an increasing burden of previous strokes were older and had more complex clinical risk profiles, and an overall higher prevalence of several comorbidities, including dementia and previous bleeding events; (3) antithrombotic strategies were marginally influenced by the number of previous strokes episodes, suggesting the need for further improvements in the secondary prevention of these patients; and (4) the risk of major cardiovascular and cerebrovascular outcomes was strongly associated with the number of previous strokes, and while the magnitude of association was numerically lower in patients treated with OAC, but we did not observe any significant interaction according to treatments or other key clinical characteristics.

Our results expand previous evidence on the natural history of patients with AF who suffered a previous stroke episode, and particularly on those with one or more episodes of stroke. Recent epidemiological studies showed comparable figures for history of thromboembolic events in patients with incident AF.^18^ The results appeared consistent with the present study which enrolled patients with a recent diagnosis of AF and suggested that some thromboembolic events may precede diagnosis of AF. Moreover, we found a high burden of clinical risk factors in those with multiple episodes of previous stroke echoing previous observations,^19^, ^20^ and our results help explain both the higher number of stroke episodes found at baseline, and the higher risk of subsequent major adverse events, beyond thromboembolic ones. Specifically, in the fully adjusted model for the prediction of future recurrent stroke, several clinical risk factors (age, hypertension, diabetes, heart failure, use of OAC) besides previous strokes were associated with an increased risk of stroke. On the other hand, an increasing body mass index was associated with decreased risk of stroke. This may suggest an “obesity paradox”, which has also been previously investigated in the context of AF in other studies,^21^, ^22^, ^23^ but further analyses are required to dissect the influence of body mass index on the risk of stroke which is out of scope for this paper. Overall, the results highlight the clinical complexity of patients with AF with previous strokes which needs to be addressed with multidimensional, holistic approach including treatment and lifestyle modification, echoing the principle of the ABC pathway.^24^, ^25^ Notably, we found that patients with 2 or more previous ischemic strokes, compared with others with less or no episodes of ischemic strokes, had higher prevalences of non‐cardiovascular comorbidities, including diabetes, chronic obstructive pulmonary disease, dementia, and previous bleeding events. These results underline the importance of the overall complexity (beyond traditional risk factors) in influencing the prognosis of patients with AF, and appear consistent with previous studies.^26^ Indeed, a previous analysis conducted on the Outcomes Registry for Better Informed Treatment registry identified, among others, chronic obstructive pulmonary disease and impaired renal functions as factors were independently associated with thromboembolism despite the use of OAC^27^; similarly, hyperlipidemia and diabetes were also associated with ischemic events in another cohort of patients with AF treated with NOAC.^28^ Taken together, these results suggest that comprehensive management of these patients, beyond adequate thromboembolic risk prevention, may be useful to mitigate the increased risk induced by the high complexity of these individuals, in accordance with current international recommendations for the management of AF.^29^, ^30^

In our analysis, we did not observe significant differences in thromboembolic risk prevention strategies of patients with AF according to the reported number of strokes at baseline. Use of OAC was broadly high, more than 80% in the overall cohort, and with approximately 60% of patients receiving an NOAC. These trends appeared consistent with the increasing uptake of OAC in recent years,^5^ led by the introduction of NOACs. Conversely, the lack of a higher use of OAC in patients with more episodes of stroke should be interpreted with caution: first, in our primary analysis we included all types of strokes, including hemorrhagic ones; moreover, we did not considered time of the most recent stroke in this analysis, and treating physicians may more cautiously use OAC in patients with recent strokes, especially hemorrhagic ones. Also, patients with previous stroke were older and with a higher prevalence of previous bleeding events (approximately 10%, compared with 5% in patients without history of stroke). The latter may also have influenced the choice of antithrombotic treatments.

While these results may suggest that further efforts should be directed to ensure that appropriate antithrombotic treatment is consistently implemented in secondary prevention of stroke in patients with AF, they also underscore the complexity and challenges in the management of these patients in real‐world practice, particularly about patients with breakthrough strokes during OAC, and for which there is still uncertainty on the best antithrombotic option.^8^, ^10^

The higher risk of all‐cause death and other outcomes that we observed in our study in patients with history of stroke confirms the very high‐risk profile of these patients, which is not limited to thromboembolic recurrences. Indeed, all‐cause mortality and cardiovascular events are consistently increased as the number of previous stroke episodes increased, and this suggest that the underlying complexity of these patients may influence their prognosis bidirectionally. These patients are often presented with complex health needs that require further management and additional strategies,^31^ accounting for the overall burden of risk factors of these patients, which also include stroke‐related disability, reduced mobility, and psychological morbidity, beyond appropriate antithrombotic therapy. These concepts have been integrated in a recent position paper of the European Society of Cardiology Council on Stroke^13^ which underscore the need to streamline an integrated and comprehensive approach to the management of stroke and associated heart disease; indeed, a management adhered to the “ABC_Stroke_” pathway was found effective in reducing the risk of stroke, major adverse cardiovascular event, and death in a retrospective analysis of the Athens Stroke Registry,^32^ suggesting that this approach may be useful to improve prognosis in these patients, similarly to what have been already demonstrated in patients with AF.^33^, ^34^, ^35^

### Strength and Limitations

This analysis is based on a large, contemporary, and multinational cohorts of patients with recent diagnosis of AF, and a long‐term follow‐up of 3 years; therefore our findings provide useful epidemiological data to improve specific knowledge on the relationship between the burden of previous stroke episodes and the natural history of patients with a recent diagnosis of AF. Notwithstanding this, we acknowledge some limitations. First, this is a retrospective analysis, and we had limited data to characterize the previous episodes of stroke in our cohort; similarly, other key factors that may influence the relationship between history of stroke, management, and long‐term prognosis may have not been available, including the timing and severity of previous strokes, acute treatments received, and residual disability. In particular, we were unable to analyze if the association between number of previous strokes and risk of major outcomes was modified by severity and/or timing of previous strokes. Patients with most recent or severe strokes may have worse prognosis, and this could have influenced our results. For these reasons, our results should be seen as exploratory and interpreted with caution. Second, although we provided multiple adjusted regression model for both antithrombotic treatment use and risk of major outcomes, we cannot exclude the contribution of residual confounders on the results observed, particularly on the use of OAC across the different groups; further studies are needed to confirm our findings, which should be again taken with caution. Third, only 1% of the total cohort of patients had 2 or more previous strokes, and this may have resulted in limited power for some of the comparisons performed, relatedly, we did not adjust our level of significance for multiple comparisons, and therefore our results—particularly on secondary outcomes – should be interpreted as exploratory.

## CONCLUSIONS

In this analysis from a contemporary and multinational real‐world registry of patients with a recent diagnosis of AF, previous stroke was found in 1 out of 10 patients, and was found associated with increased risk for subsequent adverse events during follow‐up, including all‐cause mortality. Further efforts should be directed to ensure appropriate antithrombotic treatment of these patients, and to implement additional strategies to improve prognosis, particularly in patients with multiple episodes of stroke and complex clinical risk profiles.

## Sources of Funding

The GLORIA‐AF Registry was funded by Boehringer Ingelheim GmbH. The authors are solely responsible for the design and conduct of this study, all study analyses, the drafting and editing of the manuscript, and its final contents.

## Disclosures

G.F.R. reports consultancy for Boehringer Ingelheim and an educational grant from Anthos. No fees are directly received personally. B.O. has one disclosure AstraZeneca DSMB, Consultant for Boehringer Ingelheim. T.F.C. reported honoraria for lectures from Boehringer Ingelheim, Bayer, Pfizer, and Daiichi Sankyo, outside the submitted work. M.V.H. has been receiving research grants from the Dutch Healthcare Fund, Dutch Heart Foundation, BMS‐Pfizer, Bayer Healthcare and Boehringer Ingelheim and consulting fees from BMS‐Pfizer, Bayer Healthcare and Boehringer Ingelheim to the institution. G.Y.H.L. has been consultant and speaker for BMS/Pfizer, Boehringer Ingelheim, Anthos and Daiichi‐Sankyo. No fees are directly received personally. All the disclosures happened outside the submitted work. He is a National Institute for Health and Care Research Senior Investigator and co‐PI of the AFFIRMO project on multimorbidity in AF (grant agreement No 899871), TARGET project on digital twins for personalized management of atrial fibrillation and stroke (grant agreement No 101136244) and ARISTOTELES project on artificial intelligence for management of chronic long term conditions (grant agreement No 101080189), which are all funded by the EU's Horizon Europe Research & Innovation program. The remaining authors have no disclosures to report.

## Supporting information

Tables S1–S2.
Figures S1–S4
