# Supplementary material for: Number of Previous Strokes and the Association With Clinical Outcomes of Patients With Atrial Fibrillation: Longitudinal Data From the GLORIA‐AF Registry
Source: J Am Heart Assoc. 2025 Jan 16;14(2):e038448. doi: 10.1161/JAHA.124.038448 (PMC12054405; doi:10.1161/JAHA.124.038448)
Supplement: Supplementary file 1 — Tables S1–S2. Figures S1–S4 [file JAH3-14-e038448-s001.pdf]

Supplemental Material

List of GLORIA-AF Investigators

Dzifa Wosornu Abban
Nasser Abdul
Atilio Marcelo Abud
Fran Adams
Srinivas Addala
Pedro Adragão
Walter Ageno
Rajesh Aggarwal
Sergio Agosti
Piergiuseppe Agostoni
Francisco Aguilar
Julio Aguilar Linares
Luis Aguinaga
Jameel Ahmed
Allessandro Aiello
Paul Ainsworth
Jorge Roberto Aiub
Raed Al-Dallow
Lisa Alderson
Jorge Antonio Aldrete Velasco
Dimitrios Alexopoulos
Fernando Alfonso Manterola
Pareed Aliyar
David Alonso
Fernando Augusto Alves da Costa
José Amado
Walid Amara
Mathieu Amelot
Nima Amjadi
Fabrizio Ammirati
Marianna Andrade
Nabil Andrawis
Giorgio Annoni
Gerardo Ansalone
M.Kevin Ariani
Juan Carlos Arias
Sébastien Armero
Chander Arora
Muhammad Shakil Aslam
M. Asselman
Philippe Audouin
Charles Augenbraun
S. Aydin
Ivaneta Ayryanova
Emad Aziz
Luciano Marcelo Backes
E. Badings
Ermentina Bagni
Seth H. Baker
Richard Bala
Antonio Baldi
Shigenobu Bando
Subhash Banerjee
Alan Bank
Gonzalo Barón Esquivias
Craig Barr
Maria Bartlett
Vanja Basic Kes
Giovanni Baula
Steffen Behrens
Alan Bell
Raffaella Benedetti
Juan Benezet Mazuecos
Bouziane Benhalima
Jutta Bergler-Klein
Jean-Baptiste Berneau
Richard A. Bernstein
Percy Berrospi
Sergio Berti
Andrea Berz
Elizabeth Best
Paulo Bettencourt
Robert Betzu
Ravi Bhagwat
Luna Bhatta
Francesco Biscione
Giovanni Bisignani
Toby Black
Michael J. Bloch
Stephen Bloom
Edwin Blumberg
Mario Bo
Ellen Bøhmer
Andreas Bollmann
Maria Grazia Bongiorni
Giuseppe Boriani
D.J. Boswijk
Jochen Bott
Edo Bottacchi
Marica Bracic Kalan
Drew Bradman
Donald Brautigam
Nicolas Breton
P.J.A.M. Brouwers
Kevin Browne
Jordi Bruguera Cortada
A. Bruni
Claude Brunschwig
Hervé Buathier
Aurélie Buhl
John Bullinga
Jose Walter Cabrera
Alberto Caccavo
Shanglang Cai
Sarah Caine
Leonardo Calò
Valeria Calvi
Mauricio Camarillo Sánchez
Rui Candeias
Vincenzo Capuano
Alessandro Capucci
Ronald Caputo
Tatiana Cárdenas Rizo
Francisco Cardona
Francisco Carlos da Costa Darrieux
Yan Carlos Duarte Vera
Antonio Carolei
Susana Carreño
Paula Carvalho
Susanna Cary
Gavino Casu
Claudio Cavallini
Guillaume Cayla
Aldo Celentano
Tae-Joon Cha
Kwang Soo Cha
Jei Keon Chae
Kathrine Chalamidas
Krishnan Challappa
Sunil Prakash Chand
Harinath Chandrashekar
Ludovic Chartier
Kausik Chatterjee
Carlos Antero Chavez Ayala
Aamir Cheema
Amjad Cheema
Lin Chen
Shih-Ann Chen
Jyh Hong Chen
Fu-Tien Chiang
Francesco Chiarella
Lin Chih-Chan
Yong Keun Cho
Jong-Il Choi
Dong Ju Choi
Guy Chouinard
Danny Hoi-Fan Chow
Dimitrios Chrysos
Galina Chumakova
Eduardo Julián José Roberto Chuquiure Valenzuela
Nicoleta Cindea Nica
David J. Cislowski
Anthony Clay
Piers Clifford
Andrew Cohen
Michael Cohen
Serge Cohen
Furio Colivicchi
Ronan Collins
Paolo Colonna
Steve Compton
Derek Connolly
Alberto Conti
Gabriel Contreras Buenostro
Gregg Coodley
Martin Cooper
Julian Coronel
Giovanni Corso
Juan Cosín Sales
Yves Cottin
John Covalesky
Aurel Cracan
Filippo Crea
Peter Crean
James Crenshaw
Tina Cullen
Harald Darius
Patrick Dary
Olivier Dascotte
Ira Dauber
Vicente Davalos
Ruth Davies
Gershan Davis
Jean-Marc Davy
Mark Dayer
Marzia De Biasio
Silvana De Bonis
Raffaele De Caterina
Teresiano De Franceschi

J.R. de Groot
José De Horta
Axel De La Briolle
Gilberto de la Pena Topete
Angelo Amato Vicenzo de Paola
Weimar de Souza
A. de Veer
Luc De Wolf
Eric Decoulx
Sasalu Deepak
Pascal Defaye
Freddy Del-Carpio Munoz
Diana Delic Brkljacic
N. Joseph Deumite
Silvia Di Legge
Igor Diemberger
Denise Dietz
Pedro Dionísio
Qiang Dong
Fabio Rossi dos Santos
Elena Dotcheva
Rami Doukky
Anthony D'Souza
Simon Dubrey
Xavier Ducrocq
Dmitry Dupljakov
Mauricio Duque
Dipankar Dutta
Nathalie Duvilla
A. Duygun
Rainer Dziewas
Charles B. Eaton
William Eaves
L.A Ebels-Tuinbeek
Clifford Ehrlich
Sabine Eichinger-Hasenauer
Steven J. Eisenberg
Adnan El Jabali
Mahfouz El Shahawy
Mauro Esteves Hernandes
Ana Etxeberria Izal
Rudolph Evonich III
Oksana Evseeva
Andrey Ezhov
Raed Fahmy
Quan Fang
Ramin Farsad
Laurent Fauchier
Stefano Favale
Maxime Fayard
Jose Luis Fedele
Francesco Fedele
Olga Fedorishina
Steven R. Fera
Luis Gustavo Gomes Ferreira
Jorge Ferreira
Claudio Ferri
Anna Ferrier
Hugo Ferro
Alexandra Finsen
Brian First
Stuart Fischer
Catarina Fonseca
Luísa Fonseca Almeida
Steven Forman
Brad Frandsen
William French
Keith Friedman
Athena Friese
Ana Gabriela Fruntelata
Shigeru Fujii
Stefano Fumagalli
Marta Fundamenski
Yutaka Furukawa
Matthias Gabelmann
Nashwa Gabra
Niels Gadsbøll
Michel Galinier
Anders Gammelgaard
Priya Ganeshkumar
Christopher Gans
Antonio Garcia Quintana
Olivier Gartenlaub
Achille Gaspardone
Conrad Genz
Frédéric Georger
Jean-Louis Georges
Steven Georgeson
Evaldas Giedrimas
Mariusz Gierba
Ignacio Gil Ortega
Eve Gillespie
Alberto Giniger
Michael C. Giudici
Alexandros Gkotsis
Taya V. Glotzer
Joachim Gmehling
Jacek Gniot
Peter Goethals
Seth Goldbarg
Ronald Goldberg
Britta Goldmann
Sergey Golitsyn
Silvia Gómez
Juan Gomez Mesa
Vicente Bertomeu Gonzalez
Jesus Antonio Gonzalez Hermosillo
Víctor Manuel González López
Hervé Gorka
Charles Gornick
Diana Gorog
Venkat Gottipaty
Pascal Goube
Ioannis Goudevenos
Brett Graham
G. Stephen Greer
Uwe Gremmler
Paul G. Grena
Martin Grond
Edoardo Gronda
Gerian Grönefeld
Xiang Gu
Ivett Guadalupe Torres Torres
Gabriele Guardigli
Carolina Guevara
Alexandre Guignier
Michele Gulizia
Michael Gumbley
Albrecht Günther
Andrew Ha
Georgios Hahalis
Joseph Hakas
Christian Hall
Bing Han
Seongwook Han
Joe Hargrove
David Hargroves
Kenneth B. Harris
Tetsuya Haruna
Emil Hayek
Jeff Healey
Steven Hearne
Michael Heffernan
Geir Heggelund
J.A. Heijmeriks
Maarten Hemels
I. Hendriks
Sam Henein
Sung-Ho Her
Paul Hermany
Jorge Eduardo Hernández Del Río
Yorihiko Higashino
Michael Hill
Tetsuo Hisadome
Eiji Hishida
Etienne Hoffer
Matthew Hoghton
Kui Hong
Suk keun Hong
Stevie Horbach
Masataka Horiuchi
Yinglong Hou
Jeff Hsing
Chi-Hung Huang
David Huckins
kathy Hughes
A. Huizinga
E.L. Hulsman
Kuo-Chun Hung
Gyo-Seung Hwang
Margaret Ikpoh
Davide Imberti
Hüseyin Ince
Ciro Indolfi
Shujiro Inoue
Didier Irles
Harukazu Iseki
C. Noah Israel
Bruce Iteld
Venkat Iyer
Ewart Jackson-Voyzey
Naseem Jaffrani
Frank Jäger
Martin James
Sung-Won Jang
Nicolas Jaramillo
Nabil Jarmukli
Robert J. Jeanfreau
Ronald D. Jenkins
Carlos Jerjes Sánchez
Javier Jimenez
Robert Jobe
Tomas Joen-Jakobsen
Nicholas Jones
Jose Carlos Moura Jorge
Bernard Jouve
Byung Chun Jung
Kyung Tae Jung

Werner Jung
Mikhail Kachkovskiy
Krystallenia Kafkala
Larisa Kalinina
Bernd Kallmünzer
Farzan Kamali
Takehiro Kamo
Priit Kampus
Hisham Kashou
Andreas Kastrup
Apostolos Katsivas
Elizabeth Kaufman
Kazuya Kawai
Kenji Kawajiri
John F. Kazmierski
P Keeling
José Francisco Kerr Saraiva
Galina Ketova
AJIT Singh Khaira
Aleksey Khripun
Doo-Il Kim
Young Hoon Kim
Nam Ho Kim
Dae Kyeong Kim
Jeong Su Kim
June Soo Kim
Ki Seok Kim
Jin bae Kim
Elena Kinova
Alexander Klein
James J. Kmetzo
G. Larsen Kneller
Aleksandar Knezevic
Su Mei Angela Koh
Shunichi Koide
Anastasios Kollias
J.A. Kooistra
Jay Koons
Martin Koschutnik
William J. Kostis
Dragan Kovacic
Jacek Kowalczyk
Natalya Koziolova
Peter Kraft
Johannes A. Kragten
Mori Krantz
Lars Krause
B.J. Krenning
F. Krikke
Z. Kromhout
Waldemar Krysiak
Priya Kumar
Thomas Kümler
Malte Kuniss
Jen-Yuan Kuo
Achim Küppers
Karla Kurrelmeyer
Choong Hwan Kwak
Bénédicte Laboulle
Arthur Labovitz
Wen Ter Lai
Andy Lam
Yat Yin Lam
Fernando Lanas Zanetti
Charles Landau
Giancarlo Landini
Estêvão Lanna Figueiredo
Torben Larsen
Karine Lavandier
Jessica LeBlanc
Moon Hyoung Lee
Chang-Hoon Lee
John Lehman
Ana Leitão
Nicolas Lellouche
Malgorzata Lelonek
Radoslaw Lenarczyk
T. Lenderink
Salvador León González
Peter Leong-Sit
Matthias Leschke
Nicolas Ley
Zhanquan Li
Xiaodong Li
Weihua Li
Xiaoming Li
Christhoh Lichy
Ira Lieber
Ramon Horacio Limon Rodriguez
Hailong Lin
Gregory Y. H. Lip
Feng Liu
Hengliang Liu
Guillermo Llamas Esperon
Nassip Llerena Navarro
Eric Lo
Sergiy Lokshyn
Amador López
José Luís López-Sendón
Adalberto Menezes Lorga Filho
Richard S. Lorraine
Carlos Alberto Luengas
Robert Luke
Ming Luo
Steven Lupovitch
Philippe Lyrer
Changsheng Ma
Genshan Ma
Irene Madariaga
Koji Maeno
Dominique Magnin
Gustavo Maid
Sumeet K. Mainigi
Konstantinos Makaritsis
Rohit Malhotra
Rickey Manning
Athanasios Manolis
Helard Andres Manrique Hurtado
Ioannis Mantas
Fernando Manzur Jattin
Vicky Maqueda
Niccolo Marchionni
Francisco Marin Ortuno
Antonio Martín Santana
Jorge Martinez
Petra Maskova
Norberto Matadamas Hernandez
Katsuhiro Matsuda
Tillmann Maurer
Ciro Mauro
Erik May
Nolan Mayer
John McClure
Terry McCormack
William McGarity
Hugh McIntyre
Brent McLaurin
Feliz Alvaro Medina Palomino
Francesco Melandri
Hiroshi Meno
Dhananjai Menzies
Marco Mercader
Christian Meyer
Beat j. Meyer
Jacek Miarka
Frank Mibach
Dominik Michalski
Patrik Michel
Rami Mihail Chreih
Ghiath Mikdadi
Milan Mikus
Davor Milicic
Constantin Militaru
Sedi Minaie
Bogdan Minescu
Iveta Mintale
Tristan Mirault
Michael J. Mirro
Dinesh Mistry
Nicoleta Violeta Miu
Naomasa Miyamoto
Tiziano Moccetti
Akber Mohammed
Azlisham Mohd Nor
Michael Mollerus
Giulio Molon
Sergio Mondillo
Patrícia Moniz
Lluis Mont
Vicente Montagud
Oscar Montaña
Cristina Monti
Luciano Moretti
Kiyoo Mori
Andrew Moriarty
Jacek Morka
Luigi Moschini
Nikitas Moschos
Andreas Mügge
Thomas J. Mulhearn
Carmen Muresan
Michela Muriago
Wlodzimierz Musial
Carl W. Musser
Francesco Musumeci
Thuraia Nageh
Hidemitsu Nakagawa
Yuichiro Nakamura
Toru Nakayama
Gi-Byoung Nam
Michele Nanna
Indira Natarajan
Hemal M. Nayak
Stefan Naydenov
Jurica Nazlić
Alexandru Cristian Nechita
Libor Nechvatal
Sandra Adela Negron

James Neiman
Fernando Carvalho Neuenschwander
David Neves
Anna Neykova
Ricardo Nicolás Miguel
George Nijmeh
Alexey Nizov
Rodrigo Noronha Campos
Janko Nossan
Tatiana Novikova
Ewa Nowalany-Kozielska
Emmanuel Nsah
Juan Carlos Nunez Fragoso
Svetlana Nurgalieva
Dieter Nuyens
Ole Nyvad
Manuel Odin de Los Rios Ibarra
Philip O'Donnell
Martin O'Donnell
Seil Oh
Yong Seog Oh
Dongjin Oh
Gilles O'Hara
Kostas Oikonomou
Claudia Olivares
Richard Oliver
Rafael Olvera Ruiz
Christoforos Olympios
Anna omaszuk-Kazberuk
Joaquín Osca Asensi
eena Padayattil jose
Francisco Gerardo Padilla Padilla
Victoria Padilla Rios
Giuseppe Pajes
A. Shekhar Pandey
Gaetano Paparella
F Paris
Hyung Wook Park
Jong Sung Park
Fragkiskos Parthenakis
Enrico Passamonti
Rajesh J. Patel
Jaydutt Patel
Mehool Patel
Janice Patrick
Ricardo Pavón Jimenez
Analía Paz
Vittorio Pengo
William Pentz
Beatriz Pérez
Alma Minerva Pérez Ríos
Alejandro Pérez-Cabezas
Richard Perlman
Viktor Persic
Francesco Perticone
Terri K. Peters
Sanjiv Petkar
Luis Felipe Pezo
Christian Pflücke
David N. Pham
Roland T. Phillips
Stephen Phlaum
Denis Pieters
Julien Pineau
Arnold Pinter
Fausto Pinto
R. Pisters
Nediljko Pivac
Darko Pocanic
Cristian Podoleanu
Alessandro Politano
Zdravka Poljakovic
Stewart Pollock
Jose Polo Garcéa
Holger Poppert
Maurizio Porcu
Antonio Pose Reino
Neeraj Prasad
Dalton Bertolim Précoma
Alessandro Prelle
John Prodafikas
Konstantin Protasov
Maurice Pye
Zhaohui Qiu
Jean-Michel Quedillac
Dimitar Raev
Carlos Antonio Raffo Grado
Sidiqullah Rahimi
Arturo Raisaro
Bhola Rama
Ricardo Ramos
Maria Ranieri
Nuno Raposo
Eric Rashba
Ursula Rauch-Kroehnert
Ramakota Reddy
Giulia Renda
Shabbir Reza
Luigi Ria
Dimitrios Richter
Hans Rickli
Werner Rieker
Tomas Ripolil Vera
Luiz Eduardo Ritt
Douglas Roberts
Ignacio Rodriguez Briones
Aldo Edwin Rodriguez Escudero
Carlos Rodríguez Pascual
Mark Roman
Francesco Romeo
E. Ronner
Jean-Francois Roux
Nadezda Rozkova
Miroslav Rubacek
Frank Rubalcava
Andrea M. Russo
Matthieu Pierre Rutgers
Karin Rybak
Samir Said
Tamotsu Sakamoto
Abraham Salacata
Adrien Salem
Rafael Salguero Bodes
Marco A. Saltzman
Alessandro Salvioni
Gregorio Sanchez Vallejo
Marcelo Sanmartín Fernández
Wladmir Faustino Saporito
Kesari Sarikonda
Taishi Sasaoka
Hamdi Sati
Irina Savelieva
Pierre-Jean Scala
Peter Schellinger
Carlos Scherr
Lisa Schmitz
Karl-Heinz Schmitz
Bettina Schmitz
Teresa Schnabel
Steffen Schnupp
Peter Schoeniger
Norbert Schön
Peter Schwimmbeck
Clare Seamark
Greg Searles
Karl-Heinz Seidl
Barry Seidman
Jaroslaw Sek
Lakshmanan Sekaran
Carlo Serrati
Neerav Shah
Vinay Shah
Anil Shah
Shujahat Shah
Vijay Kumar Sharma
Louise Shaw
Khalid H. Sheikh
Naruhito Shimizu
Hideki Shimomura
Dong-Gu Shin
Eun-Seok Shin
Junya Shite
Gerolamo Sibilio
Frank Silver
Iveta Sime
Tim A. Simmers
Narendra Singh
Peter Siostrzonek
Didier Smadja
David W. Smith
Marcelo Snitman
Dario Sobral Filho
Hassan Soda
Carl Sofley
Adam Sokal
Yannie Soo Oi Yan
Rodolfo Sotolongo
Olga Ferreira de Souza
Jon Arne Sparby
Jindrich Spinar
David Sprigings
Alex C. Spyropoulos
Dimitrios Stakos
Clemens Steinwender
Georgios Stergiou
Ian Stiell
Marcus Stoddard
Anastas Stoikov
Witold Streb
Ioannis Styliadis
Guohai Su
Xi Su
Wanda Sudnik
Kai Sukles
Xiaofei Sun
H. Swart
Janko Szavits-Nossan

Jens Taggeselle
Yuichiro Takagi
Amrit Pal Singh Takhar
Angelika Tamm
Katsumi Tanaka
Tanyanan Tanawuttiwat
Sherman Tang
Aylmer Tang
Giovanni Tarsi
Tiziana Tassinari
Ashis Tayal
Muzahir Tayebjee
J.M. ten Berg
Dan Tesloianu
Salem H.K. The
Dierk Thomas
Serge Timsit
Tetsuya Tobaru
Andrzej R. Tomasik.
Mikhail Torosoff
Emmanuel Touze
Elina Trendafilova
W. Kevin Tsai
Hung Fat Tse
Hiroshi Tsutsui
Tian Ming Tu
Ype Tuininga
Minang Turakhia
Samir Turk
Wayne Turner
Arnljot Tveit
Richard Tytus
C Valadão
P.F.M.M. van Bergen
Philippe van de Borne
B.J. van den Berg
C van der Zwaan
M. Van Eck
Peter Vanacker
Dimo Vasilev
Vasileios Vasilikos
Maxim Vasilyev
Srikar Veerareddy
Mario Vega Miño
Asok Venkataraman
Paolo Verdecchia
Francesco Versaci
Ernst Günter Vester
Hubert Vial
Jason Victory
Alejandro Villamil
Marc Vincent
Anthony Vlastaris
Jürgen vom Dahl
Kishor Vora
Robert B. Vranian
Paul Wakefield
Ningfu Wang
Mingsheng Wang
Xinhua Wang
Feng Wang
Tian Wang
Alberta L. Warner
Kouki Watanabe
Jeanne Wei
Christian Weimar
Stanislav Weiner
Renate Weinrich
Ming-Shien Wen
Marcus Wiemer
Preben Wiggers
Andreas Wilke
David Williams
Marcus L. Williams
Bernhard Witzenbichler
Brian Wong
Ka Sing Lawrence Wong
Beata Wozakowska-Kaplon
Shulin Wu
Richard C. Wu
Silke Wunderlich
Nell Wyatt
John (Jack) Wylie
Yong Xu
Xiangdong Xu
Hiroki Yamanoue
Takeshi Yamashita
Ping Yen Bryan Yan
Tianlun Yang
Jing Yao
Kuo-Ho Yeh
Wei Hsian Yin
Yoto Yotov
Ralf Zahn
Stuart Zarich
Sergei Zenin
Elisabeth Louise Zeuthen
Huanyi Zhang
Donghui Zhang
Xingwei Zhang
Ping Zhang
Jun Zhang
Shui Ping Zhao
Yujie Zhao
Zhichen Zhao
Yang Zheng
Jing Zhou
Sergio Zimmermann
Andrea Zini
Steven Zizzo
Wenxia Zong
L Steven Zukerman

Table S1 – Multivariable Cox Regression for revealing the predictors of future recurrent stroke

	aHR (95% CI)	p-value
**Total number of previous strokes**		
No Previous Stroke	Ref.	
1 Previous Stroke	2.07 (1.66 – 2.58)	<0.001*
≥2 Previous Strokes	3.33 (2.04 – 5.42)	<0.001*
**Age**		
<65	Ref.	
65 to <75	1.48 (1.13 – 1.94)	0.004*
>=75	2.31 (1.77 – 3.00)	<0.001*
**Female**	1.09 (0.92 – 1.29)	0.334
**AF type**		
Paroxysmal AF	Ref.	
Persistent AF	1.28 (1.06 – 1.54)	0.011*
Permanent AF	1.42 (1.07 – 1.89)	0.016*
**Regional recruitment**		
Europe	Ref.	
North America	1.14 (0.9 – 1.43)	0.275
Asia	1.38 (1.09 – 1.74)	0.006*
Other	0.79 (0.54 – 1.15)	0.218
**Previous bleeding**	1.20 (0.87 – 1.66)	0.273
**BMI**	0.98 (0.96 – 0.99)	0.005*
**Hypertension**	1.42 (1.14 – 1.78)	0.002*
**Diabetes**	1.21 (1.00 – 1.48)	0.056
**HF**	1.24 (1.01 – 1.52)	0.037*
**PAD**	1.09 (0.69 – 1.72)	0.715
**CAD**	1.12 (0.91 – 1.39)	0.287
**OAC**	0.69 (0.56 – 0.85)	0.001*

* depicts statistically significant results at p<0.05 level. aHR= adjusted Hazard Ratio; CI= Confidence Intervals; Ref.= Reference; AF= Atrial Fibrillation; BMI= Body Mass Index; HF=Heart Failure; PAD= Peripheral Artery Disease; CAD= Coronary Artery Disease; OAC= Oral Anti-Coagulant

Table S2 – Multiple Cox Regressions on Major Outcomes according to the number of previous strokes – Sensitivity Analysis restricted on patients with ischemic stroke

	No Previous Stroke (n=19165)	1 Previous Stroke (n=1719)	≥2 Previous Strokes (n=201)
**Primary Outcome**			
All-Cause Death			
IR [95%CI]	3.2 [3.0-3.3]	4.8 [4.2-5.5]	8.2 [6.0-11.1]
aHR [95%CI]	Ref.	1.49 [1.29-1.71]*	2.47 [1.81-3.37]*
**Secondary Outcomes**			
CV Death			
IR [95%CI]	1.1 [1.0-1.2]	1.5 [1.2-1.9]	3.1 [1.8-5.1]
aHR [95%CI]	Ref.	1.35 [1.05-1.73]*	2.72 [1.63-4.56]*
MACE			
IR [95%CI]	2.2 [2.0-2.3]	3.8 [3.3-4.4]	6.8 [4.7-9.6]
aHR [95%CI]	Ref.	1.65 [1.40-1.96]*	3.00 [2.11-4.28]*
Thromboembolism			
IR [95%CI]	1.1 [1.1-1.2]	3.3 [2.7-3.8]	5.2 [3.5-7.8]
aHR [95%CI]	Ref.	2.53 [2.09-3.06]*	4.07 [2.71-6.09]*
Major Bleeding			
IR [95%CI]	1.2 [1.1-1.3]	1.4 [1.1-1.8]	2.0 [0.9-3.6]
aHR [95%CI]	Ref.	1.17 [0.90-1.52]	1.09 [0.52-2.30]

* depicts statistically significant results at $p<0.05$ level. aHR= adjusted Hazard Ratio; CI= Confidence Intervals; IR= Incidence Rate, Ref.= Reference.

Figure S1 – Multiple Logistic Regression on OAC and NOAC vs. VKA use in patients according to the number of previous stroke

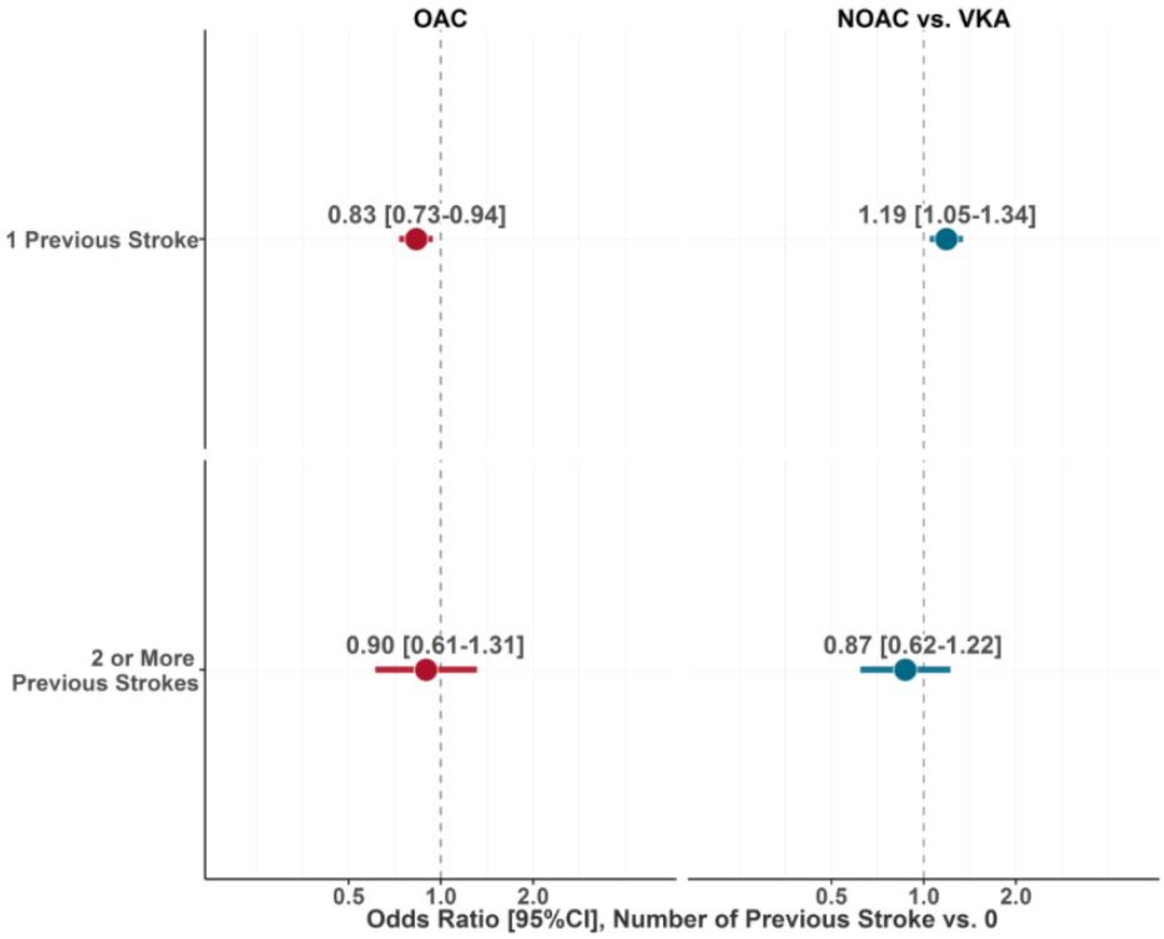

NOAC= Non vitamin-K antagonist Oral Anticoagulant; VKA= Vitamin K Antagonist

Figure S2 – Use of Antithrombotic according to the number of previous stroke – Sensitivity Analysis restricted on patients with ischemic stroke

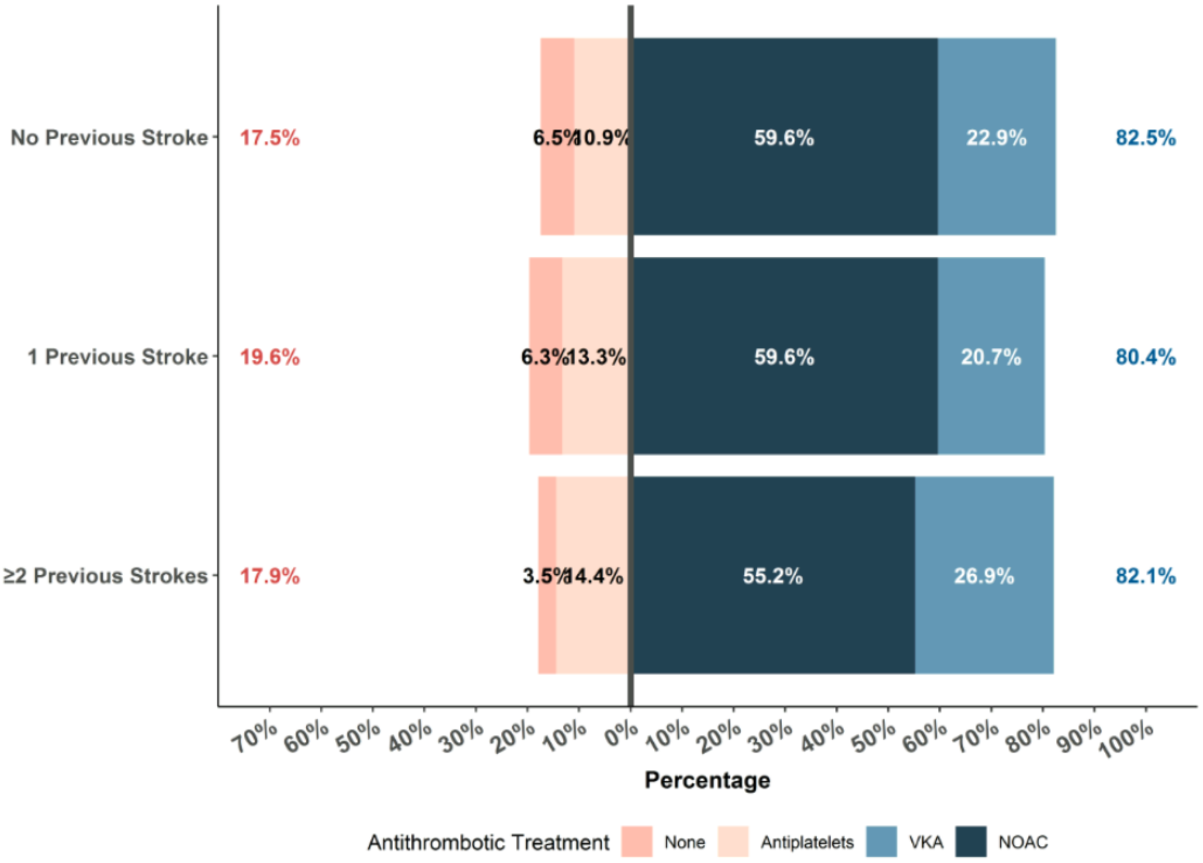

NOAC= Non vitamin-K antagonist Oral Anticoagulant; VKA= Vitamin K Antagonist

Figure S3 – Multiple Logistic Regression on OAC and NOAC vs. VKA use in patients according to the number of previous stroke - Sensitivity Analysis restricted on patients with ischemic stroke

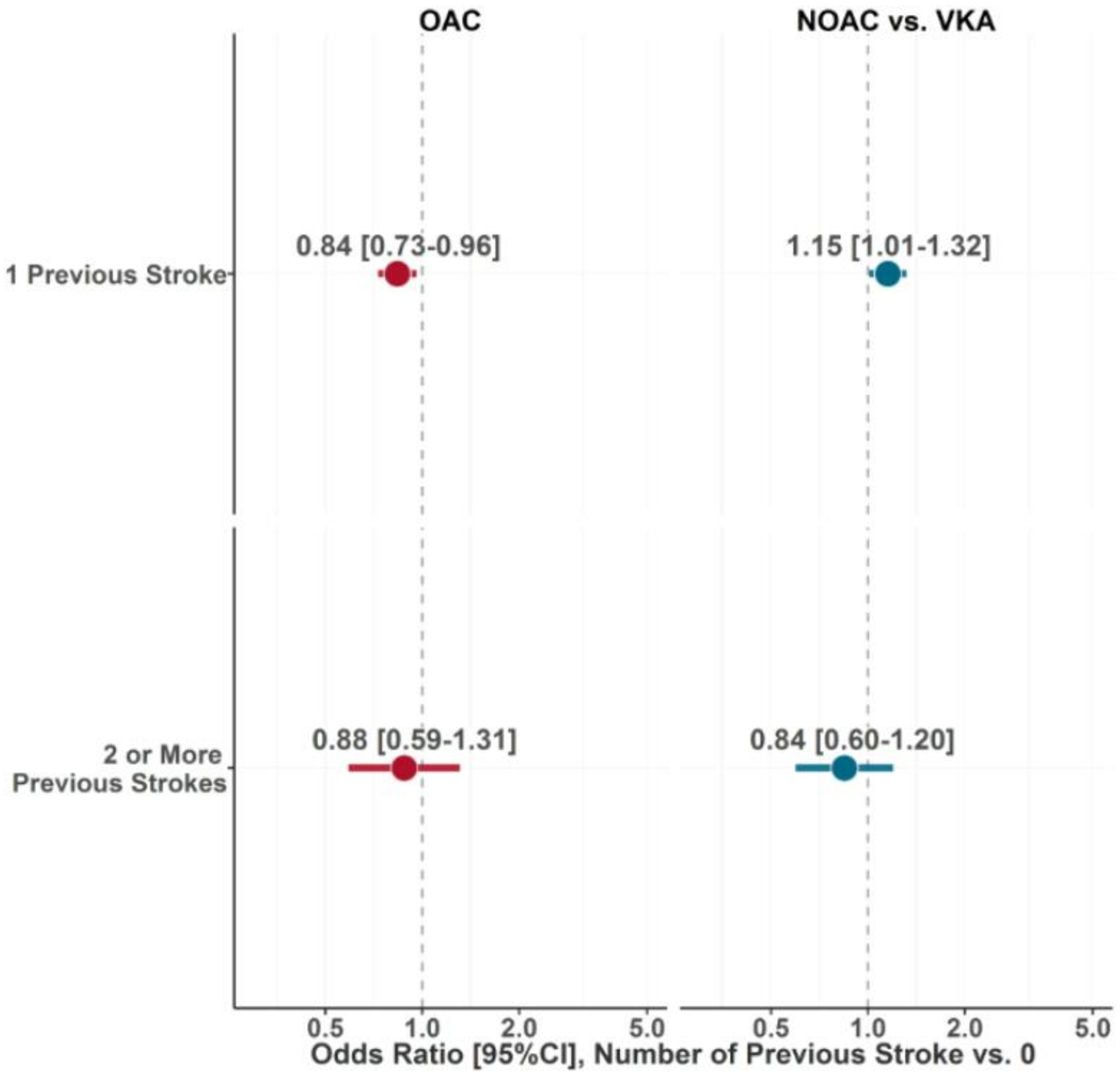

NOAC= Non vitamin-K antagonist Oral Anticoagulant; VKA= Vitamin K Antagonist

Figure S4 – Survival Curves according to the number of previous stroke for the primary outcome of all-cause death - Sensitivity Analysis restricted on patients with ischemic stroke

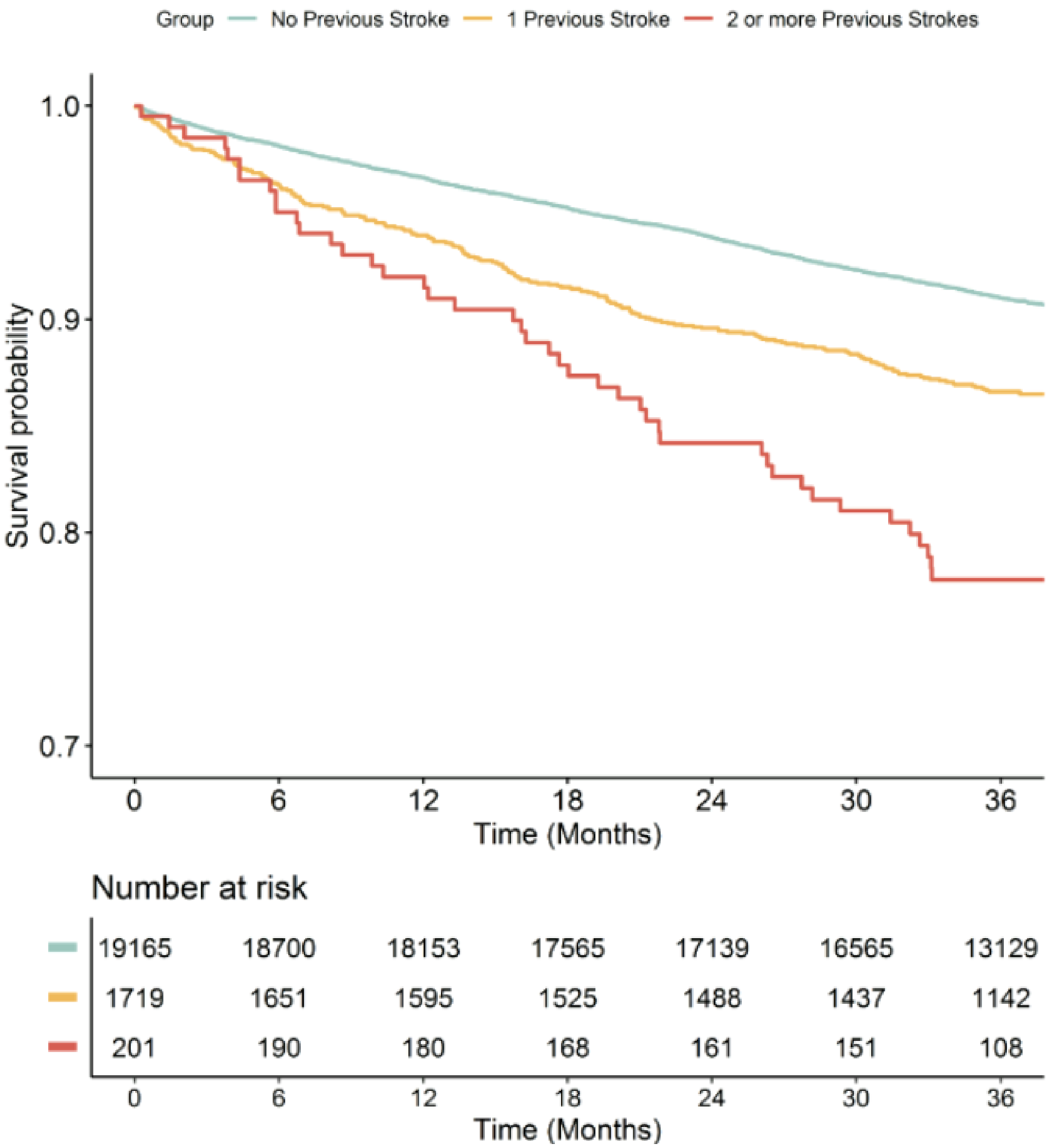

Log-Rank p<0.001
